# A user-friendly system for identifying the optimal insertion direction and to choose the best pedicle screws for patient-specific spine surgery

**DOI:** 10.1016/j.heliyon.2024.e26334

**Published:** 2024-02-16

**Authors:** Alfonso Magliano, Francesco Naddeo, Alessandro Naddeo

**Affiliations:** Department of Industrial Engineering, University of Salerno, Fisciano, SA, Italy

**Keywords:** Spine surgery, Pedicle arthrodesis, Optimal insertion direction, Surgical Template, Standard screw, Surgical planning, Neurosurgery

## Abstract

**Background and objective:**

Many diseases of the spine require surgical treatments that are currently performed based on the experience of the surgeon. The basis of this study is to deliver an automatic and patient-specific algorithm able to come to the aid of the surgeons in pedicle arthrodesis operations, by finding the optimal direction of the screw insertion, the maximum screw diameter and the maximum screw length.

**Results:**

The paper introduce an algorithm based on the reconstructed geometry of a vertebra by 3D-scan that is able to identify the best introduction direction for screw and to select, from commercial and/or personalised databases, the best screws in order to maximize the occupation of the bone while not intersecting each other and not going through the walls of the pedicle and the bounds of the vertebral body. In fact, for pedicle arthrodesis surgery, the incorrect positioning of the screws may cause operating failures, an increase in the overall duration of surgery and, therefore, more harmful, real-time X-ray checks. In case of not availability on market, the algorithm also suggests parameters for designing and manufacturing an ‘ad hoc’ solution. The algorithm has been tested on 6 vertebras extracted by a medical database. Furthermore, the algorithm is based on a procedure through which the surgeon can freely choose the entering point of the screw (based on his/her own experience and will). A real patient vertebra has been processed with almost 400 different entering point, always giving a feedback on the possibility to use the entering point (in case of unavailability of a good trajectory) and on the individuation of the right trajectory and the choose of the better screws.

**Conclusions:**

In very recent bibliography, several papers deal with procedure to screw’ trajectory planning in arthrodesis surgery by using Computer Aided surgery systems, and some of them used also modern methodologies (KBE, AI, Deep learning, etc.) methods for planning the surgery as better as possible. Nevertheless, no methodologies or algorithm have been still realized to plan the trajectory and choose the perfect fitting screws on the basis of the patient-specific vertebra. This paper represents a wind of novelty in this field and allow surgeons to use the proposed algorithm for planning their surgeries. Finally, it allows also the easy creation of a customized surgical template, characterized by two cylindrical guides that follow a correct trajectory previously calculated by means of that automatic algorithm generated on the basis of a vertebra CAD model for a specific patient. The surgeon will be able to set the template (drilling guides) on the patient's vertebra and safely apply the screws.

## 1 Introduction

In recent years, the development of increasingly complex technologies has given a great help in achieving even better results in different areas, especially the medical one. In this paper, the authors create an automatic and patient-specific algorithm which can detect the optimal direction of the screw insertion needed for the pedicle arthrodesis surgery, following that patient-specific vertebra reproduced as a computer-aided design (CAD) model, obtained through a reverse engineering operation [1]. The algorithm also finds the optimal screws that must be inserted in the pedicle in terms of diameter and length, and it makes a comparison between the obtained results and the existing screws available on market. From this procedure, a customized surgical template can be realized, to help the surgeon during the screws' placement. The template is realized with two cylindrical hollow guides to facilitate the directional drilling. The guides' direction will match the optimal screws’ insertion directions previously calculated by the algorithm [2]. The combination of an automatic algorithm and a surgical template, both customized for the specific patient who needs an arthrodesis surgery for spine stabilization, leads to excellent results in screw positioning accuracy, reducing the overall duration of the procedure, reducing the number of times the patient is exposed to X-rays, improving the intervention safety and the overall results quality [3,4].

## 2 State of the art

For posterior stabilization of the spine, a frequently used procedure is screw placement in vertebrae pedicles [5]. The attempt to surgically block more vertebral segments has always created problems for the orthopaedic surgeon. In surgery, this fusion is accomplished by placing a bone graft and/or bone graft substitute, that bridges the vertebrae, allowing new bone to grow into the space [6]. Thus, this procedure can be used to treat pain caused by spinal motion or instability.

In cases of disorders and diseases where results cannot be obtained using corrective devices or physiotherapy, the pedicle arthrodesis surgery is needed. The experience of the surgeon and the specialization of the centres where the arthrodesis of the lumbar, thoracic, cervical vertebrae is performed is fundamental to ensure the success of the surgery. The main risk is that the graft does not heal or that it undergoes dislocation (more frequent in the absence of implants). Moreover, it is possible to damage the blood vessels directed towards the legs, intestines or ureter and, in the case of men, retrograde ejaculation (for interventions near the L5 and S1 vertebrae). Other possible risks include hernias, damage to the diaphragm, kidneys, nerve roots or spinal cord, bleeding, and infections [7,8].

The arthrodesis surgery is made by using peduncular screws that penetrate the vertebrae through the structures that connect the vertebral body with the posterior arch. These screws laterally across the canal that contains the spinal cord (so they are fully contained in the pedicles): this explains the delicacy and difficulty of this technique.

The screws are made of titanium (or steel), they are threaded and they have a mobile head to allow rotations and support any vertebral effort [9]. Once the screws have been inserted and their placement has been verified, fixing bars must be realized and then, they must be slightly curved by means of a special clamp bender. The bars are placed and fixed with a special stud that geometrically connects them to the head of the screw to ensure its stability. In the end, an additional verification X-ray is performed [10,11]. Despite spinal fixations with pedicle screws are widely used nowadays to provide spine stability and correct spinal deformity [12,13] for pedicle arthrodesis surgery, two critical factors must be addressed: screws must be applied correctly and exposure to harmful radiation must be avoided or limited.

The incorrect positioning of the screws may cause operating failures that lead to subsequent re-operations, an increase in the overall duration of surgery and, therefore, more harmful, real-time X-ray checks [2]. The variability of pedicles’ geometries and orientation, their small size, as well as the existence of nearby nerve roots and vascular structures can cause problems for screw placement itself [3]. Calculating the best trajectory for pedicle screw insertion is crucial to avoid all the above problems [14,15]. Lots of studies have been conducted on how trajectories should be calculated and pedicle screws should be planned [[16], [17], [18], [19], [20]].

There are so many ways of having a spine arthrodesis surgery.

**Fig. 1:**
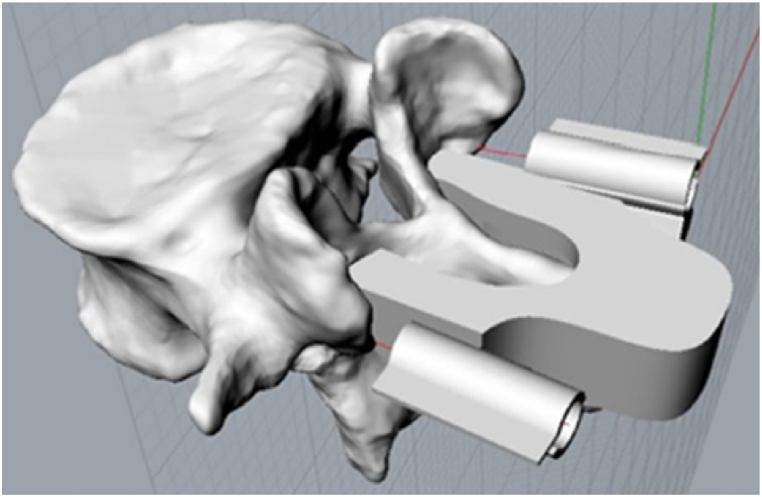
Lumbar vertebra coupled with a template. The template is custom-made for the specific arthrodesis operation. It will be adapted on the vertebra (suction cup effect) to facilitate the surgery.

• *Classic surgery*: it is the conventional freehand technique. In this case, the surgeon's experience is fundamental. No robotic machinery is used during the operation. The classic surgery is the most difficult type of operation, and it is high-time consuming [21].
• *Robot*-*assisted surgery*: medical robotics in modern orthopaedic surgery. Robot-assisted techniques are increasingly applied to spine surgery to reduce the rate of screw misplacement [22]. However, controversy about the superiority of robot-assisted techniques over conventional freehand techniques remains [23,24]. Of course, the surgeon can't be totally replaced.
• *Navigation systems*: similar to the robot-assisted surgery, advanced imaging navigation systems [25] are really expensive and they are not available in the majority of the hospital globally.
• *Template surgery*: a template is used to help the surgeon for the screw's insertion. To avoid vertebrae's breakthrough problems, a solution consists of designing a ‘custom made’ template for spinal surgery, customized and optimized for the individual patient, aimed at the directional drilling of the vertebrae. The templates are characterized by two guides with hollow cylindrical geometry, with the function of guiding the tip of the surgical drilling tool so that the hole is realized in the position chosen by the surgeon in the pre-surgery phase (see Fig. 1). Although the area to be operated is clean because the surgical team has previously ‘skeletonized’ the area, blood spills are unavoidable during the entire phase of the operation. This causes instability for the positioning of the drilling mask on the vertebral surface due to the presence of superficial and deep muscle tissues and bundles that affect the area to be operated. To overcome this problem, several holes are made. These holes are made on adhesion surface at the laminae that run along the entire length of the body of the template until passing through the smaller section of the same. Thanks to the presence of these holes, the blood spills that are not sucked by the ‘cannula’ used by surgeons, will flow through them. This strategy allows a greater adhesion and stability of template on the vertebra, creating a ‘suction effect’ [2,10].

The proposed algorithm falls in the template arthrodesis surgery. In fact, the algorithm is realized to find the optimal direction of the screw insertion, the maximum screw diameter and the maximum screw length, so that a customized surgical template can be obtained through a rapid manufacturing process, helping the surgeon because he only needs to place this patient-specific template on the selected area and put the screws (obtained from the algorithm and compared to the existing catalogues) inside the special guides designed for specific vertebrae [[2], [3], [4], [5],^26^].

This algorithm evaluates the optimal screw insertion direction and screw's diameter. These parameters will correspond to the template hole's direction and diameter. This matching will provide optimal results for thepatient-specific surgery. The results of this application have been evaluated through an evaluation table from Ref. [1] applied to 8 vertebrae (5 real patient and 3 from database) studied by two neurosurgeons (see Table 1).

**Table 1:** Table used by the two neurosurgeons to test the algorithm's reliability.

Passage of the screw for pedicle center	1		2		3		4		5	
**Screws alignment with vertebral plate**	1		2		3		4		5	
**Screws axis convergence in the sagittal plane**	1		2		3		4		5	
**Screws interpenetration in vertebral surface**	CRITICS (>4 mm)		RELEVANT (From 3 to 4 mm)		MODERATE (From 2 to 3 mm)		SLIGHT (<2 mm)		ABSENT	
**Has the algorithm met the expectation?**	1	2	3	4	5	6	7	8	9	10

## 3 Methodology

The proposed algorithm is realized to work with any vertebra of any patient.^1^ The idea is to use it to help the surgeon in finding the optimal screws’ insertions directions and their diameters, so that a specific template can be realized for that specific subject.

The algorithm is generalized to deal with cervical, lumbar and thoracic vertebrae. This patient-specific algorithm is a modification of another patented algorithm [1] used for several procedures, designs and validations [3,4,26]. The modifications have been introduced to make the algorithm faster, optimized and readable by different CAD programs. It has been written in Python programming environment [27], using several modules. Some steps of the procedure are equal, while in other cases, the approach is completely different.

When the algorithm runs, it must receive as input the patient-specific Stereo Lithography interface format (.stl) CAD file of the vertebra, appropriately segmented into homogeneously distributed triangles, obtained through a reverse engineering operation. The patient's vertebra is acquired by CT scan (Computed Tomography). Using a medical image processing commercial software, the scan is segmented and rebuilt in a 3D workspace environment. The 3D reconstruction of the vertebrae is imported into the CAD software (for example, Rhinoceros), used for 3D modelling of sculptured surfaces (free form). In Rhinoceros, all geometric entities are represented by NURBS (Non-Uniform Rational B-Splines) [2,28].

**Fig. 2:**
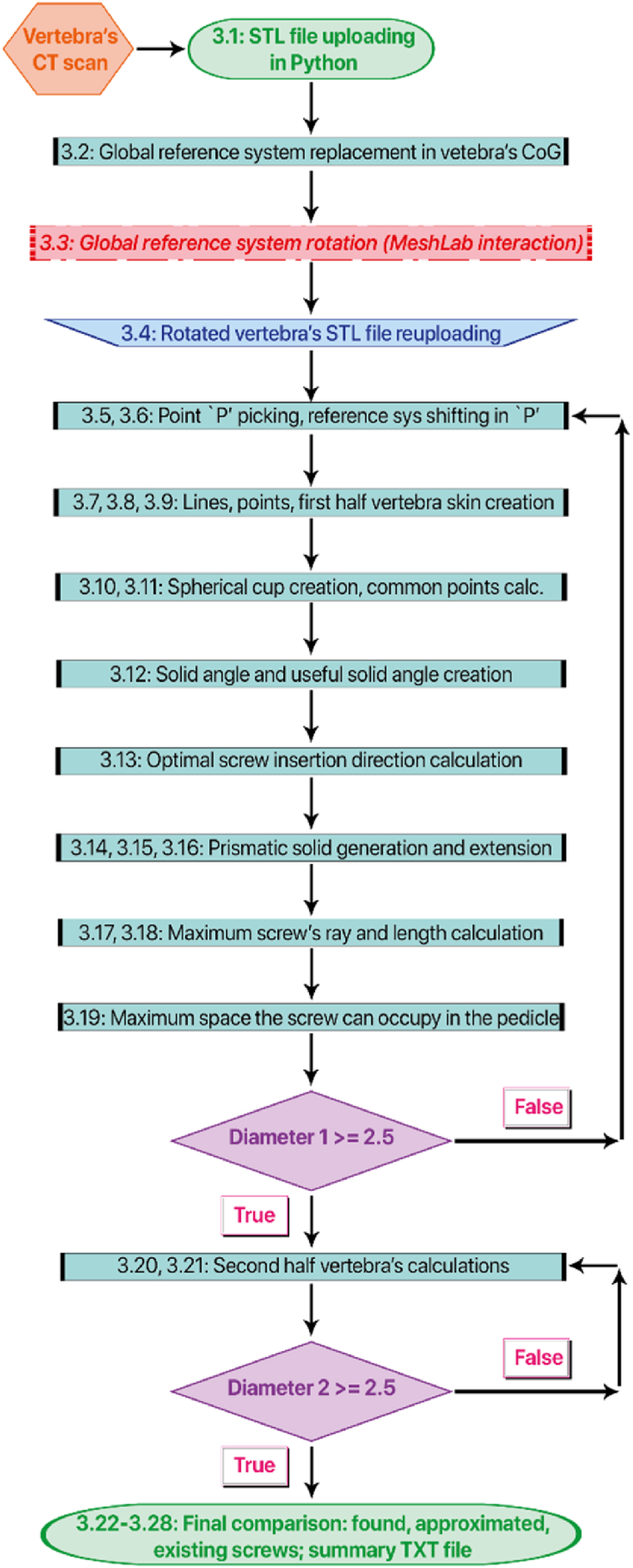
Algorithm's flowchart. It outlines all the new algorithm's steps. The *green-water* blocks represent all the steps needed to get the results. The *purple* blocks contain the conditions corresponding to the real existing screw; they allow the comparisons. The *orange* block characterizes all the external steps made before starting the algorithm (all the mandatory elements to start the process that are not made in the algorithm itself). The *red* block corresponds to the interaction with an external software (in this case MeshLab, used for generalizing the vertebra's placement in the 3D space). The *blue* block is the one that takes as input the vertebra from MeshLab and make it useful/useable for the algorithm. The *green* blocks represent the start (vertebra's uploading in the Python Console) and the end (comparisons) of the algorithm. (For interpretation of the references to colour in this figure legend, the reader is referred to the Web version of this article.)

The new algorithm consists of 28 steps (the previous. stl vertebra's input file achievement is not included here; the algorithm starts by entering the. stl vertebra's file already obtained). The first 20 steps are related to the first half vertebra. In fact, during the procedure, the vertebra will be divided into two halves, so that the two screws can be found separately. From the medical point of view, to divide any human body bone into two halves (right and left halves) the sagittal plane is needed. The 21st step is the repetition of the 20 previous steps, but now applied to the 2nd half vertebra. The remaining steps (from 22nd to 28th) are realized to get the final surgery's results and the comparison between found and existing screws on catalogues (see Fig. 2).

The algorithm is interactive: the surgeon (or an engineer or a technician) interacts with the code, which will lead to the final results step by step. The code asks which file must be studied, what point must be the entry point, how many screws exist for that specific point of that patient-specific vertebra etc.

Moreover, before the screw's entry point choice, the code interacts with MeshLab [29]. This interaction is needed to facilitate the vertebra's placement in the 3D space.

### 3.1 STL file uploading in Python

The first thing to do is to import the. stl file in Python. To have a generalized algorithm, the vertebra's.stl file is manually imported in Python, by writing the vertebra's name (.stl) in the Python Console.

### 3.2 Global reference system displacement in the vertebra center of gravity

This step is made to facilitate the identification of the three fundamental planes (sagittal, coronal and.transverse). Moreover, this command makes the visualization easier in the virtual environment.

After this translation, the vertebra is automatically saved.

### 3.3 Global reference system rotation

This step is made in MeshLab. In this open-source system for processing and editing 3D triangular meshes, the surgeon (or the engineer or the technician) can rotate the vertebra as wanted. This step is fundamental because, to reach the desired results, the global x axis must go through the vertebra, dividing it into two halves, the y axis must be the normal vector of the sagittal plane (so that this plane is unambiguously defined by this two axes) and the z axis is, by construction, the up-going axis. MeshLab automatically opens when the axes are translated in the vertebra's center of gravity. The file in MeshLab is the one previously saved at 3.2.

Alongside MeshLab, a. txt file (formerly created and placed in the same folder of the code) appears: it contains all the information needed to rotate in the correct way the vertebra in MeshLab. Until the vertebra is fully rotated and MeshLab is closed, the algorithm won't go on. Before closing MeshLab, the vertebra must be saved. After this rotation, the Python algorithm goes on.

### 3.4 Rotated vertebra's STL file uploading in Python

The previously rotated vertebra must be uploaded in Python. To generalize the algorithm, the vertebra is uploaded manually, after the usage of MeshLab (3.3).

### 3.5 Screw's entry point picking

**Fig. 3:**
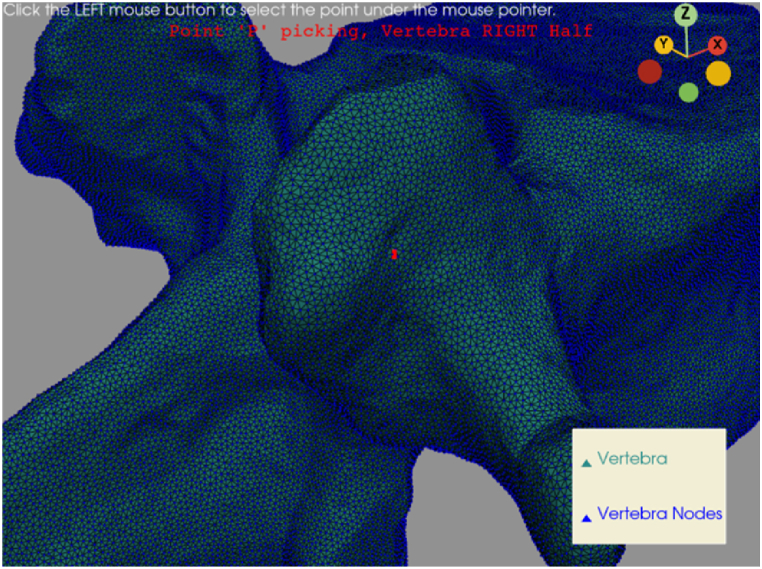
Entry point picking. In this way, the surgeon can select the entry point for the screw (red point). (For interpretation of the references to colour in this figure legend, the reader is referred to the Web version of this article.)

The only manually defined point is the screw's entry point, by clicking on the vertebra's 3D. It will match one of the vertebra's nodes. This step aims at simulating the surgeon point choice (see Fig. 3). In fact, from this point, the surgeon can insert the peduncular screw and start the vertebra stabilization operation. The Python module used to accomplish this step is *PyVista* [30]. This module will be the most used for this procedure, due to its versatility and simplicity. It also allows to plot images (the following images are obtained with PyVista) and to create human-computer interactions.

### 3.6 Global reference system replacement in the chosen entry point

This step is needed to succeed in the future algorithm steps. For example, to create solid angles starting from the entry point, the global reference system must be centred in the entry point.

### 3.7 Straight line ‘L’, point ‘C’, ‘D’, ‘F’, ray ‘PC’ definition

**Fig. 4:**
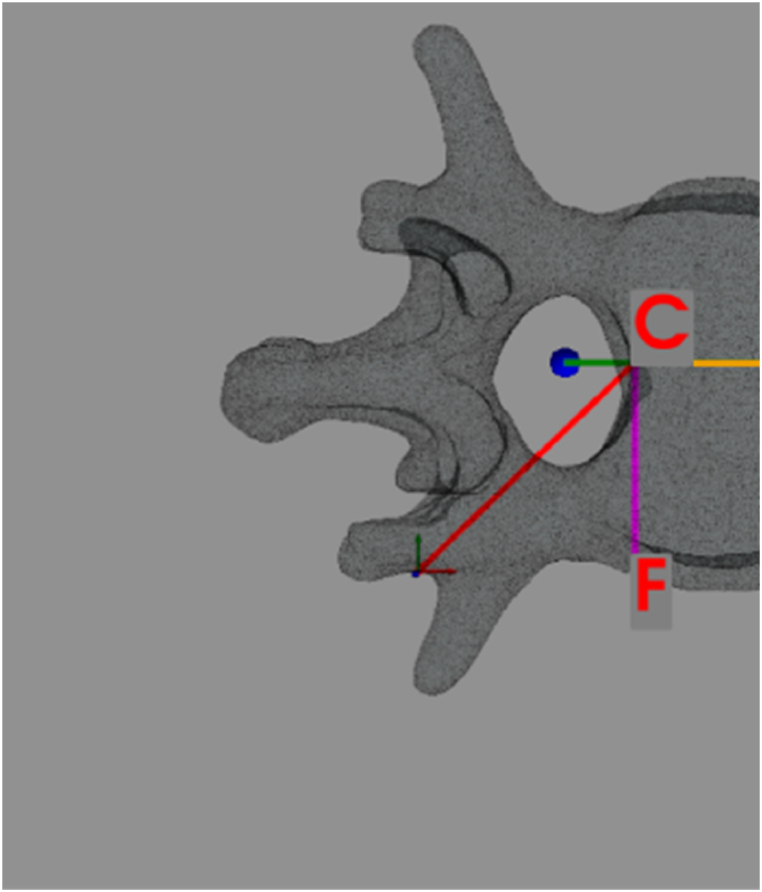
Construction lines for planes' creation (for the first half vertebra, right side).

These construction elements (see Fig. 4) are needed to get the vertebra's division into two halves.

From these elements, sagittal plane, transverse plane, coronal plane and half vertebra skin can be created. In particular.

• The straight line ‘L’ is obtained starting from the center of gravity, ending in a point with same center of gravity y, but different x and z coordinate. Straight line ‘L’ will intersect the vertebra in two points: the first one is the point ‘C’, the second one is the point ‘D’. Straight line ‘CD’ can be made by linking these two points. Due to the vertebra's geometry, it can happen the straight line won't intersect the vertebra itself: in this case, a new starting point is created with an ‘*if loop*’ (this point is the centroid moved in z direction of ±5 to ensure the line ‘L’ intersects all types of vertebrae: lumbar, thoracic, cervical).
• The straight line ‘CF’ is obtained by rotating by 90° the ‘CD’ straight line around z axis. From here, point ‘F’ is extracted (it is the last point of the ‘FC’ straight line). This information is needed to get the sagittal plane (PyVista uses the plane normal vector to create a plane; the sagittal plane normal vector will match ‘CF’).
• Ray ‘PC’ is obtained by linking point ‘P’ and point ‘C’. This ray will be used to create a spherical cup, needed to obtain the optimal screw insertion direction.

It is important to underline that both straight line ‘L’ and straight line ‘CF’ creation operation can be made because the system has been previously rotated. The rotation is fundamental. In fact, wrong rotations will lead to wrong line and point creations and algorithm's failure.

### 3.8 Sagittal, coronal and transverse planes definition

Thanks to the elements defined at 3.7, the sagittal, coronal and transverse planes can be created.

The *sagittal* plane divides body parts (so every bone, vertebrae, entire body etc.) into right and left; it is created using the normal plane vector (obtained from the straight line ‘CF’) and the plane center (the choice is arbitrary, in this case point ‘C’ is chosen as plane center). The normal sagittal vector will match -y direction for the 1st half vertebra and +y direction for the 2nd half vertebra (but this 2nd normal vector will be created later in the code).

The *coronal* plane divides the body parts into front (or anterior) and back (or posterior); it is obtained by rotating the sagittal plane by 90° around z. This plane will be important later, when it is necessary to control if the screw intersect the anterior vertebra's part and the sagittal plane.

The *transverse* plane divides the body parts into up and down; it is obtained by rotating the sagittal plane by 90° around x. For this application, transverse plane will not be used, but it is still represented.

It is redundant, but mandatory, to remember that these rotations applied to create the coronal and transverse planes from the sagittal plane are realized because the global reference system has been previously rotated in MeshLab.

### 3.9 First half vertebra's skin creation

This step is accomplished by using another Python module, similar to PyVista: *Trimesh*. It allows an easier mesh manipulation and analysis [31].

Due to some problems during the computation, Trimesh solution seemed to be the way that fitted best.

### 3.10 Spherical cup creation

The spherical cup is created to obtain the optimal direction. It has no medical aim.

**Fig. 5:**
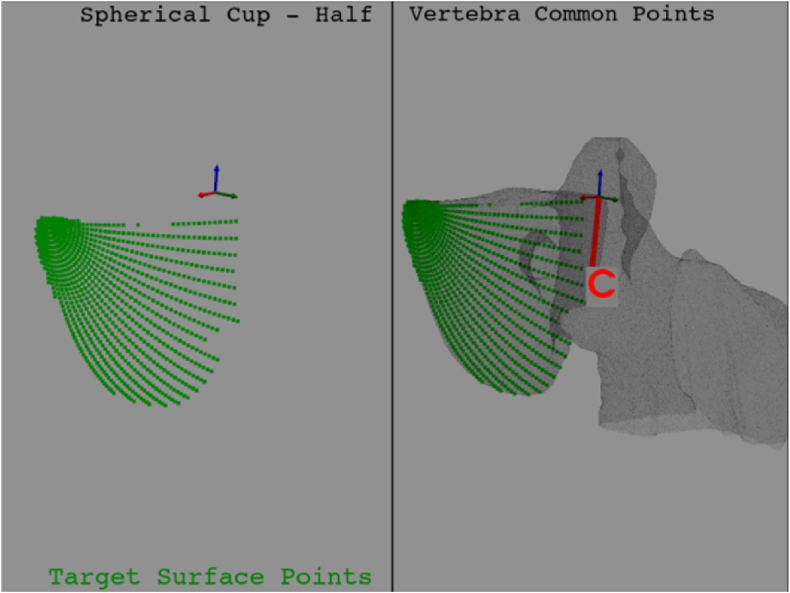
Target surfaces' points. These points are the result of the half vertebra - spherical cup intersection. They are needed for the solid angle creation.

From the spherical cup, a target surface can be obtained (by intersecting the half vertebra with spherical cup). Each point of the target surface (see Fig. 5) will be linked to the entry point, so that the solid angle can be created. The number of the spherical cup points depends on the spherical coordinates values: θ, which domain is [0, 360] and φ, which domain is [0, 70]. These values are calculated from the starting reference system's placement, with a resolution of 70 points for both longitudinal (θ) and latitudinal (φ) directions. The points should be equally spaced; however, the results will be almost the same.

### 3.11 Spherical cup - half vertebra common points calculation

This step is mandatory to extract the points of the target surface. These points (see Fig. 5) will be the ending points of the solid angle.

### 3.12 Solid angle and useful solid angle creation

• *Solid angle*: it is obtained by linking all the spherical cup - half vertebra common points (3.11) to the entry point. This solid angle is made up of several straight lines; not all these lines are entirely contained in the vertebra pedunculus.

**Fig. 6:**
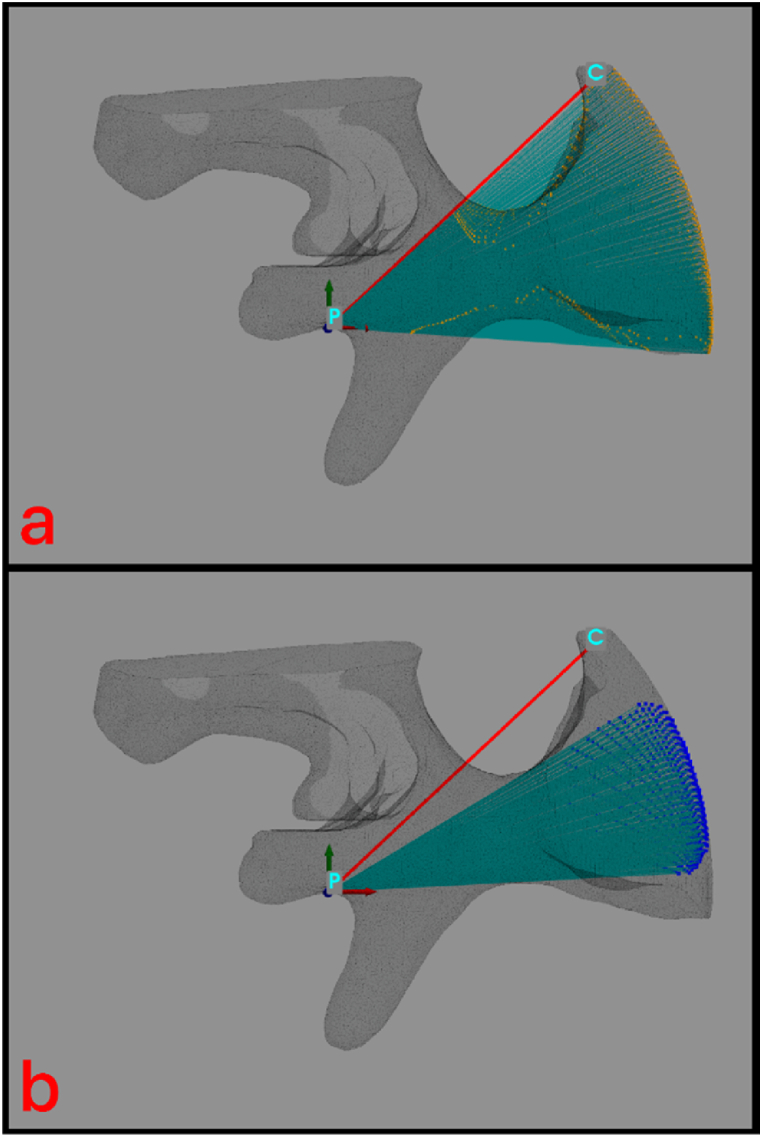
**(a)**: Solid angle. The yellow points are the intersection points between the solid angle and the half vertebra's body; **(b)**: Useful solid angle. The blue points are the useful target surface's points. (For interpretation of the references to colour in this figure legend, the reader is referred to the Web version of this article.)

With PyVista multi ray trace command, the solid angle is generated (see Fig. 6a) and the solid angle - half vertebra intersection points are calculated. But only the lines entirely contained in the pedunculus are needed to perform the maximum screw diameter and length: useful solid angle must be realized.

• *Useful solid angle*: from the spherical cup - half vertebra common points (3.11) another ray trace command (like the one used to obtain the solid angle) is performed, but now in a *for loop.* This PyVista command creates a series of vectors, each one containing from two to several points (depending on if there are intersections between the solid angle's lines and the half vertebra): if the vector contains only two points, it is kept (these two points are the entry point and one of the spherical cup - half vertebra common points); while if the vector contains more than two points, it is not kept because that means the line passing through these multiple points intersects the half vertebra (and the line is not good to create the useful solid angle).

Now, a new vector is created: it will contain only the first element of each two-points vector (so this new vector will contain only the ‘*useful’* spherical cup - half vertebra common points able to generate the useful solid angle (see Fig. 6b)).

At the end, the useful solid angle is generated, by connecting the entry point with all the points contained in the new vector. The useful solid angle is the cone which the screw will pass through.

### 3.13 Optimal screw insertion direction calculation

**Fig. 7:**
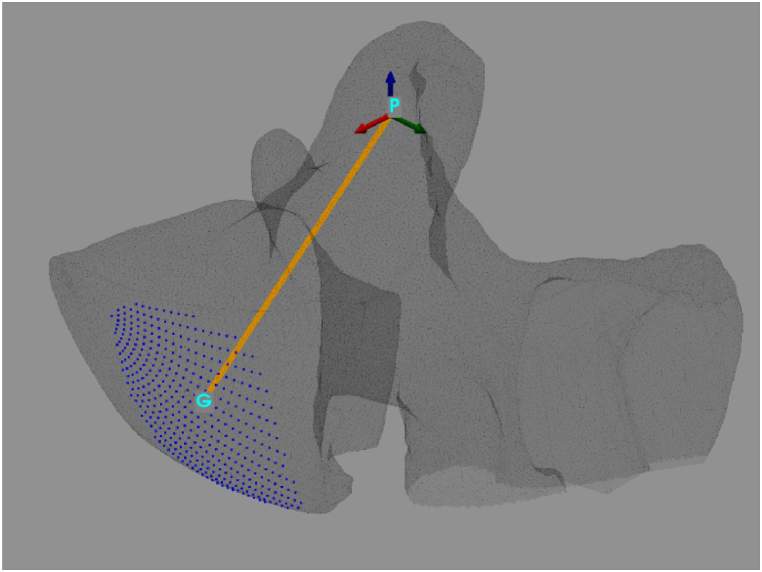
Optimal screw's insertion direction.

The optimal screw insertion direction is obtained [2] by linking the screw entry point to the centroid (point ‘G’) of the ‘useful’ spherical cup - half vertebra common points (3.12, see Fig. 7).

### 3.14 First and second planes perpendicular to the optimal direction creation

The first plane is needed to start with the generation of a prismatic solid, which will contain the final screw. This plane's center is the entry point.

The second plane is parallel to the first one and it plays the same role of the first plane, but this new plane is created to avoid problems during the projection of the ‘useful’ spherical cup - half vertebra common points (3.12).

### 3.15 Prismatic solid generation

The prismatic solid is the solid into which the cylinder (representing the final screw) will fall. This solid must be entirely contained into the half vertebra pedunculus, so that the cylinder will do the same too.

To get the prismatic solid, several steps were followed.

a) Projection of the useful spherical cup - half vertebra common points on the second plane.
b) Connection between useful spherical cup - half vertebra common points and their projection. With PyVista multi ray trace command, connection lines and intersection's points will be generated.
c) Splitting of the intersection's points previously obtained into ‘cup’ and ‘base’ points. When PyVista ray trace command is performed in a for loop, a series of vectors, each one containing from two to several elements, is created (procedure similar to solid angle - useful solid angle creations, 3.12). Then, only the vectors with two elements are kept and, at the end, these vectors can be separated: the first one will contain the points on the target surface while the second one will contain the points on the other sides of the half vertebra.
d) Connection between the intersection's points previously obtained. The resulting lines will be all within the vertebra's pedunculus, but they do not create a prismatic solid due to the vertebra's geometry. Some of them, instead, will be the useful prismatic solid generators.
e) Useful prismatic solid generators. To get the useful lines to generate a prismatic solid so that it is contained in the pedunculus, lines with length equal or greater than 1.75 times the half of the optimal direction line length are taken, eliminating all the other lines. This value is chosen because of the geometry that characterize the vertebrae.
f) Prismatic solid base creation. The useful prismatic solid generators are lines with a starting and an ending point. By extracting all the initial points and projecting them over the first plane perpendicular to the optimal direction (3.14), the prismatic solid base is obtained. This base will contain the base of the cylinder (which simulates the peduncular screw).

### 3.16 Algorithm extension to the whole first half vertebra

Until this point, all the calculations were conducted with the target surface reference. The screw will not stop at the target surface, but it will continue until the end of the half vertebra; that means a prismatic solid perfectly containing the screw's body must be generated (this new prismatic solid is a prolongation of the previous prismatic solid (3.15,e). This new prismatic solid was not created because this step would have been useless; only the optimal screw insertion direction was extended instead.

### 3.17 Maximum screw ray and diameter calculation

From the prismatic solid base (3.15,f), the perimeter, the edges and their joining points are extracted. To get the maximum screw radius, all these joining points are linked to the screw entry point: the shortest connecting line will represent the maximum possible screw radius. This line is multiplied by a safety coefficient (in this case, it is chosen as 0.925) to make sure the screw falls correctly within the vertebra pedunculus. From the radius, diameter calculation is trivial. The radius is needed to create the screw; in fact, the PyVista command uses the radius. The diameter is necessary because the international catalogues define the spine arthrodesis screws with length and diameter, indeed.

### 3.18 Maximum screw length calculation

If the screw's length is equal to the extended optimal direction line (3.16), the screw's volume will intersect the half vertebra (in its front part or in the sagittal plane or both). Intersections with both half vertebra anterior part and sagittal plane must be avoided. To let the screw fall into the half vertebra body without touching anything (so that the screw is perfectly contained in the half vertebra pedunculus) a simple loop is created. Firstly, the maximum obtainable cylinder (with length equal to the extended optimal direction) is created. Then, this cylinder will be reduced step by step (step = 0.0025 mm) until a non-intersecting cylinder will be obtained. For this application, the 10th cylinder which does not touch the half vertebra is chosen (for safety reason). To obtain the intersection between the half vertebra and the cylinders and verify the maximum available path for the screw, the coronal plane is needed (3.8). In fact, the intersection is realized between the quarter vertebra and the cylinder instead of the half vertebra. If the half vertebra is used, the program will always give back an intersection between the half vertebra and the cylinder because the entry point will always represent an intersection. The quarter vertebra's creation is realized by means of Python module Trimesh, with the same procedure applied in 3.9 for the half vertebra's creation.

### 3.19 Maximum space the screw can occupy within the half vertebra

**Fig. 8:**
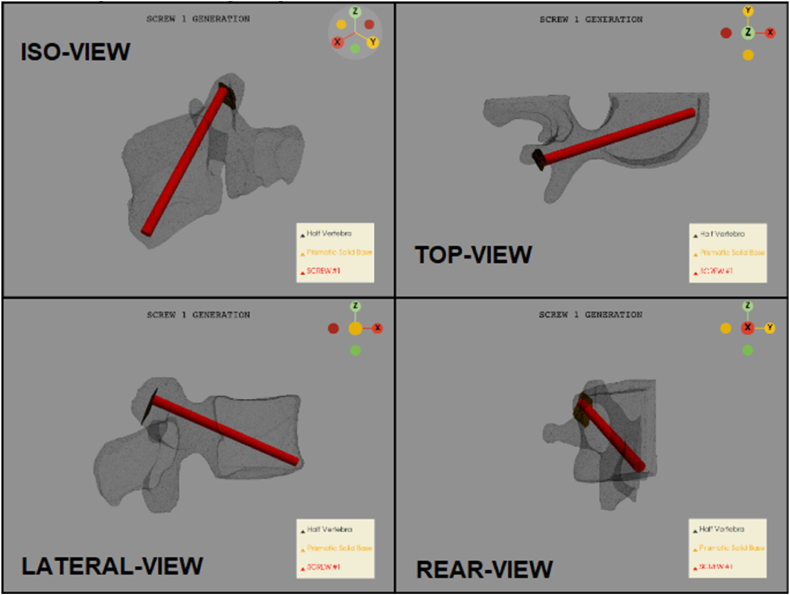
Maximum available space. This image is related to the first screw (right side). Top left: ‘iso’ view; Top right: ‘xy’ view; bottom left: ‘xz’; bottom right: yz’.

From the previous evaluated parameters, the cylinder representing the screw can be generated. This is the maximum space available in the half vertebra's pedicle for a screw (see Fig. 8).

### 3.19.1 Repetition of the previous 19 steps if the found screw is not available

After the achievement of the maximum available path for the screw's insertion, a check of the obtained diameter (3.17) is made. This is mandatory because one of the algorithm's goals is to find the existing screws that better fit the patient-specific arthrodesis surgery. From literature, the minimum existing screws' diameter available on the market is equal to 2.5 mm. Therefore, an if loop is implemented in the algorithm: if the calculated diameter (3.17) is greater than 2.5 mm, the algorithm jumps at the Step 20 of the procedure, whereas if the resulting diameter is smaller than 2.5 mm, the algorithm restarts from the entry point picking (3.5) because it means the previous picked entry point gave back a screw that do not exist (and the patient cannot be operated). The procedure continues until a new diameter (3.17) and a new maximum available screw's path (3.19) is found. In this way, the surgeon has another opportunity to pick an entry point, probably close to the previous wrong one, and re-start the algorithm. To avoid the surgeon re-picks the same previous point, and to remember where the previous point was, the entry point re-pick also shows the previous wrong picked point. This re-pick can be made 4 times at most. At the fifth attempt, the algorithm goes on and it starts with 3.20 and with the second half vertebra procedure (3.21).

### 3.20 Screw's parameters approximation

The screw's parameters in terms of diameter (3.17) and length (3.18) previously obtained will be highly probably different from the screw's parameters available on catalogues.

The diameter is approximated to always get a value divisible by 5 (because of the existing screws and because stronger approximation will make the calculation too safe): for example, from a value equal to 4,89 mm the algorithm gives back a value of 4,5 mm, while from 4,05 mm it gives back 4,0 mm.

The length is truncated to the nearest smaller value: for example, from a value of 53,49 mm, a value of 53 mm is retrieved. The diameter approximation is obtained in 3.17, while the length approximation is obtained in 3.19. In this step, these values are invoked only. They are needed to make the final comparison with the existing screws.

### 3.21 Repetition of the previous steps for the second half vertebra

The procedure is now applied to the second half vertebra (sagittal left side). The steps are the same, with some differences because now the calculations are made for the other half of the vertebra.

The differences are the following one.

• *3.5: second entry point picking* (entry screw point for the 2nd screw).
• *3.6: global reference system shifting in this second entry point.*
• *3.7: ‘CD’ and ‘PC’ lines recalculation because of global reference system shifting in the second entry point.* Line ‘CH’ calculation (not present in 3.7 of the right half vertebra: this line is ‘CF’ line substitution) because from this line is possible to generate the new sagittal normal vector. In fact, the sagittal plane is the same, but the normal vector for the second half vertebra goes in the opposite direction compared to the normal vector of the first half vertebra: with the opposite normal vector, the second half vertebra (left side) skin can be obtained.
• *3.9: Second half vertebra skin creation.*

### 3.22 Two screws results’ union

This step allows to have a first complete vision of the patient-specific arthrodesis results. The plotted screws are the ones with the found parameters instead of the approximated ones. From this step until the end of the procedure, the global reference system is changed: now the axes are centred in the point ‘C’. That means, to make the comparisons and visualizations in the future next steps, all the plotted elements have been translated as point ‘C’ is now the new global reference system origin.

This choice is applied because point ‘C’ is placed on the sagittal plane, and this allows a good vertebra visualization.

### 3.23 Graphic comparison between ‘found’ and ‘approximated’ screws

The approximated screws increase the insertion safety. In addition, it is now possible to compare the found results with the approximated screws, both graphically and numerically.

### 3.24 Graphic comparison between ‘approximated’ and ‘existing’ screws

It can be possible that the obtained approximated screws are not present in existing catalogues: the algorithm works (because it ends up with results), but no screws are available on market.

The complete catalogue of the existing screws is uploaded in Python, then the comparison takes place. The uploaded catalogue is realized in Excel by unifying several catalogues found online: Nexon [32], FireBird [12], EgiFix [33], AF Medical [34], GS Medical [35], Expedium® [36], DePuySynthes [37], Kanghui [38], ulrich Medical [39], Medacta [40], BMR®Extremities [41], Neo Medical [42], Z-Medical [43] and GPC Medical USA [44]: more than 2800 screws are used for the comparison.

About the comparison.

• Comparison between the approximated diameter and all the existing screws' diameters. If there are available screws with this diameter, the comparison continues.
• Comparison between all the existing screws found from the previous comparison (all the screws with the same diameter of the approximated one) and the approximated length: only the screws with the first length smaller or equal then the approximated one will be taken.

The comparison tables will show if both screws (for both first and second half vertebra) are available for that patient-specific vertebra operation.

The tables include diameter and length of the available screw (both in millimetres) but also the reference number of the screw, the type of screw and the manufacturing company. The information about the type of screw is important because, in some cases, the algorithm can give positive results, but the subject cannot be operated because the screw cannot be applied for that vertebra (for example, the vertebra to be operated is a cervical vertebra, but available screws with the right parameters can be only used for lumbar vertebrae).

Another information the tables give back is the number of screws available for that specific operation (how many screws with that diameter and length can be used for that entry point ‘P’). If the screws are not available on market, it will be printed in the Python Console.

After the comparison, both tables information is stored in separated Excel files.

### 3.25 Choice of the screw with the smallest diameter and length

After the comparison with existing screws, it is possible to choose the screw with the smallest diameter and length. This happens because screws are often sold in pairs and so it is not possible to choose different screws for one vertebra's surgery. The algorithm is able to choose a combination of parameters that minimize both diameter and length: for example, it can happen that the smallest diameter is related to the first screw, but the smallest length is related to the second screw. In this case, the algorithm finds in the catalogue the screw with these minimized parameters. So, from the catalogue, the screw with the smallest diameter and length is taken: the screw can be one of the two previously obtained ones or the combination of the two smallest parameters that characterize the two previously obtained screws.

### 3.26 Final graphic and numeric comparison: found, approximated and existing screws

This paragraph shows a numerical and graphic comparison between found, approximated and existing screws. The numerical comparison shows how the procedure steps from found values of diameters and lengths to the existing values of them, resulting from the smallest values’ combination obtained at 3.25. The graphic comparison is the graphical plot of the numerical results. This plot is needed to have a more practical idea of how the algorithm worked and to have a visual impact of where the screws will be placed.

### 3.27 Final comparison: useful and useless entry points, useful screws

This comparison is useful to check the difference between the maximum available path which the screws can be inserted in and to see which entry point for that patient-specific arthrodesis surgery are good or wrong. In particular, in case of procedure repetition (3.19.1) the plot will show a points’ cloud, where the red points represent the wrong entry points while the green represent the correct one (with or without procedure repetition, the two green points are the correct entry points for right half and left half vertebra).

Moreover, if the algorithm arrives at the fifth attempt without finding a correct screw's diameter, the plot will show this last point as green: anyway, this is the correct entry point if that not existing screw would exist.

### 3.28 Final comparison: main surgery's results storage in TXT file

The last step of the procedure is a summary of the whole patient-specific surgery. When the algorithm finishes its calculation, after the last plot, a. txt file opens. In this. txt file, all the main information of that specific operation is stored: in this way, the surgeon has a complete and general framework of the arthrodesis surgery just simulated.

## 4 Results

This procedure can replace the classic procedure for the identification of the optimal screws’ insertion direction. In fact, the traditional procedure involves longer time (stress and significant bleeding for the patient, stress and increased probability of error for the surgeon), longer exposure to X-rays for both the patient and the medical team and longer time for entry screw point identification.

With the application of this procedure to the template surgery, all the above problems can be drastically reduced [2].

The algorithm is realized to be user-friendly, intuitive and as simple as possible.

The interactions human-Python and human-MeshLab are easy and driven by. txt files that appear when necessary to provide the instructions to execute the code without mistakes and any stop that can undermine the final results and the algorithm's reliability. Time resolution was optimized in order to make it as shorter as possible. However, it strictly depends on two steps of the procedure: 3 and 18.

The algorithm's aim is to obtain the optimal screw insertion direction, generate the screw for that direction and make a comparison with the existing screws on market, every time an entry point is chosen, for both right and left half vertebra, for any vertebra that must be operated.

So, in terms of results, from the 22nd to the 28th step, the surgeon can understand what happens for that patient-specific arthrodesis surgery.

The main results from this algorithm are.

• First half vertebra's screw's diameter and length, maximum values.
• Second half vertebra's screw's diameter and length, maximum values.
• First half vertebra's screw's diameter and length, real existing values.
• Second half vertebra's screw's diameter and length, real existing values.
• Comparison between found, approximated and existing screws (if they screws exist, 3.26).
• What happens when the screws are not available on the market (3.26).
• Graphic comparison between maximum available screws' path and existing/not existing screws (3.27).
• Graphic plot of the correct/wrong entry points (3.27).

Part of the results have already been discussed in the procedure, from 3.22 to 3.28. Here, a more detailed results’ analysis will be conducted, in particular for the 3.26 and 3.27.

The *3.26* gives back the final comparison, for both existing and non-existing screws. That means that, if for the first half vertebra no screws are found in 5 attempts, the algorithm goes on to the second half vertebra and, as results of the first half vertebra, it takes the results of the last attempt (the same for the second half vertebra; if the first half vertebra gives good results but the second half vertebra gives bad results). As consequence of this, the choice of the smallest screws' couple will result in a non-existing screws’ couple, because for one of the two halves, after 5 attempts, no available screws were found.

**Fig. 9:**
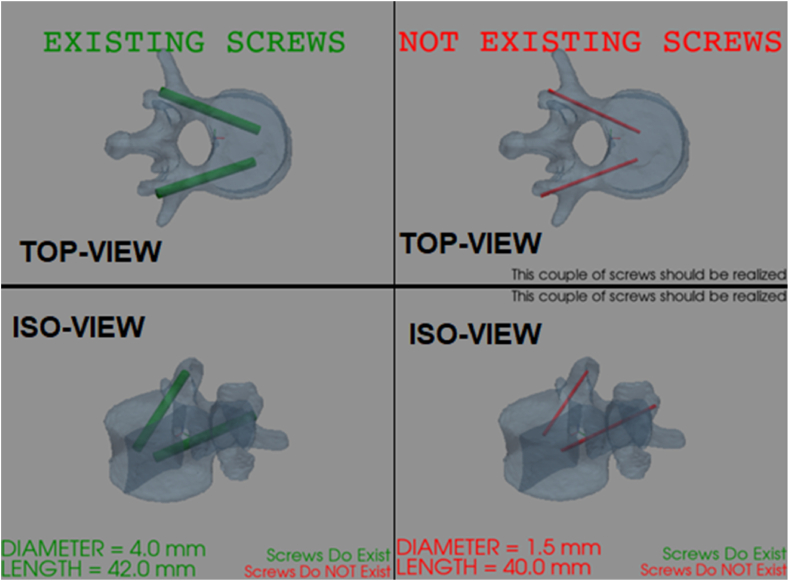
Results' dynamic plots. The figure shows two different attempts (to highlight how the plot is dynamic). Left side: the screws' couple exists. Right side: the screws' couple does not exist.

**Fig. 10:**
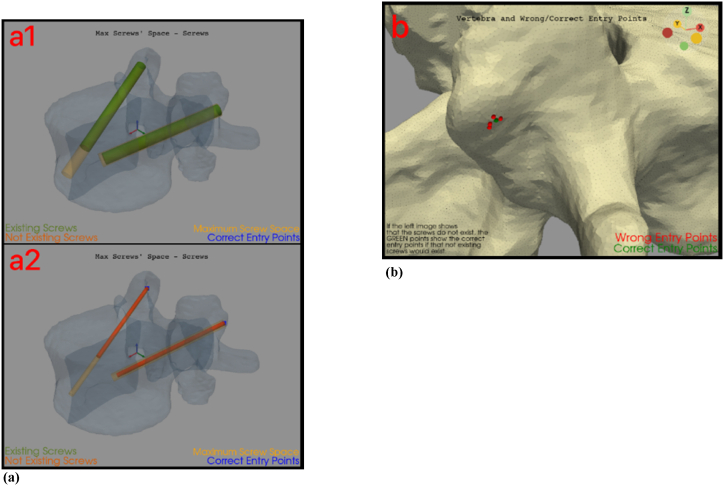
(a): Maximum screws' available path. The figure shows two different attempts (to highlight how the plot is dynamic). Top, a1: the screws' couple exists. Bottom, a2: the screws' couple does not exist. Fig. 10: (b): Points' cloud. This cloud of points represents the points the surgeon had chosen for that patient-specific surgery. The red points are the ones which did not give back an existing screw. (For interpretation of the references to colour in this figure legend, the reader is referred to the Web version of this article.)

Then, the plot of the 3.26 is a dynamic plot, that changes depending on if the screws are available or not (see Fig. 9). The *3.27* is the graphic comparison between maximum available screws' path and existing/not existing screws. This plot is dynamic too. In fact, the resulting screws in the maximum available path are dark orange-coloured if they do not exist yet. When the screws' couple exist, they will be green-coloured. In both cases, the maximum screws’ available path is yellowish (see Fig. 10,a).

The 3.27 is also characterized by another plot, which represents the correct/wrong screws' entry points. If the procedure finds both screws at firsts attempts, then this plot will show only two green points (correct entry points), one on the right side of the vertebra and the other one on the left side. Otherwise, if the procedure, for first, second or both halves, will be repeated (3.19.1), a points' cloud will be plotted, where red points are the wrong entry points, while the green are the correct ones (see Fig. 10,b).*4.1. Algorithm's test*.

The algorithm's creation has been conducted using a lumbar vertebra as input, given ‘ad hoc’ for this project. To check the algorithm's reliability, different. stl files have been used.

In particular, the input vertebrae are: 4 lumbar vertebrae (3 real cases); 3 thoracic vertebrae (2 real cases); 1 cervical vertebra. The. stl files used in this application have been found online [[45], [46], [47], [48]].

### 4.1 A case study: the lumbar vertebra

Random entry points for both first and second half vertebra were chosen to test the algorithm. This solution is used to have a general idea of how the algorithm works and what kind of results it can give in terms of errors, screws' availability and diameters’ percentage frequency appearance. Results are obtained through Python and Excel programs. As already explained in the paragraph 3.24, the catalogue has been created in Excel from online catalogues; then, it is uploaded in Python and the “search and compare” algorithm can be run.: the algorithm searches and selectin the uploaded catalogue, automatically, all the screws with a diameter equal to the approximated one. After this selection, another algorithm search and select, among the previously found screw with the right diameter, the first screws whose length is lower or equal to the approximated one. At the end of the process the selected screws, if any, will be taken.

**Fig. 11:**
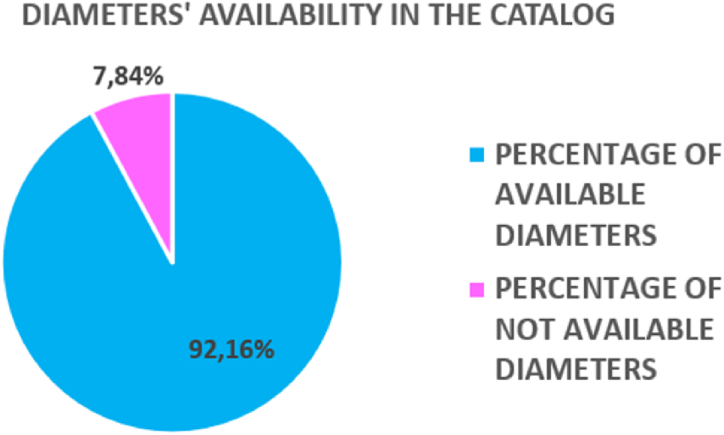
Case study's results: diameters' availability.

About the diameters’ availability in the catalogue, 92,2% of the diameters are available (see Fig. 11). The remaining 7,8% of the diameters is not available because of only one reason: the final obtained diameters are smaller than 2,5 mm (smallest available diameter in the catalogue).

Related to the diameters' percentage frequency appearance, diameters in between 0,5 mm and 2,0 mm are less frequent than diameters from 2,5 mm to 6,5 mm. From the algorithm's attempts, two reasonable results came out.

• 85,9% of times both screws are available, while 14,1% of times at least one screw is not available: that means the algorithm works, but the patient cannot be operated because of the nonexistence of the screw(s). In this case, the screw should be created for that patient-specific arthrodesis surgery.
• When both screws are available, only 10,3% of times the screws are equal (so equal diameter and length). For the remaining 89,7%, the screws are different (in terms of diameter and length, or only length). This low value of equal screws' couples percentage can be explained. The sagittal plane (3.8) is generated starting from the volume center of gravity of the vertebra. Depending on where the center of gravity is placed (how the vertebra's mass is distributed in the space), the available space for the screw's insertion cannot be homogeneous.

### 4.2 Short virtual trial by neurosurgeons

**Fig. 12:**
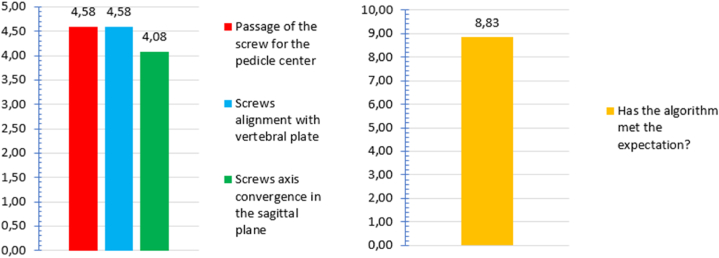
(a, left): Short virtual trial's results. **(b, right):** Global neurosurgeons algorithm's evaluation.

The algorithm evaluates the optimal screw insertion direction and screw's diameter, making a comparison with the existing screws available on market. To have more reliable results, the algorithm was given to two neurosurgeons. They evaluated the algorithm behaviour through the table previously shown in Table 1, testing 8 vertebrae: 5 of them where real vertebrae while 3 were taken from database. The results are collected in Fig. 12 (a and b).

a) In a scale from 1 to 5, passage of the screw for the pedicle center, screws alignment with vertebral plate and screws axis convergence in the sagittal plane were evaluated: the first two parameters obtained 4,58/5 while the last one 4,08/5.
b) The algorithm's expectation was evaluated on a 1/10 scale, reaching 8,83/10.

Moreover, both neurosurgeons certified the absence of interpenetration in vertebral surface.

### 4.3 Algorithm's limitations

The proposed algorithm is subject to four weak limitations, which require an optimization to improve the reliability. Anyway, the algorithm works well.

These limitations are.

1. 3.3: vertebra rotation. This step is realized with the interaction with MeshLab, but an optimization of the code can ensure the rotation within the Python environment. This change will not improve the code accuracy, but it will make it easier to be used for the surgeon/engineer/technician.
2. 3.10: spherical cup creation. PyVista generates a cup with not equally spaced points. An equally spaced points cup should be used, despite it will not bring important changes in the global algorithm working.
3. 3.15: prismatic solid generation. For the useful prismatic solid generators creation, another method can be found to optimize the algorithm. The one here proposed still works well.
4. 3.18: maximum screw length and cylinder calculation. This part of the code works well, but it can be still improved by optimizing the for loops.

In addition, the algorithm has got similarities with [18], describing a computer-assisted tool for preoperative pedicle screw placement planning in CT images, designed with respect to the vertebral shape and structure. They both evaluate the screws’ trajectory, but it also considers the bone density and intra and extra peduncular surgery.

## 5 Future developments

This algorithm must be considered as a starting point for future improvements.

Future developments can be.

• After the cylinder generation, check what happens if the entry point is changed, while the obtained cylinder is kept for the comparison.
• What happens if the surgeon chooses the point in a completely different zone of the vertebra, for example, on another surface.
• Find or develop more screws (screw with diameters smaller than 2.5 mm, for the cases when the algorithm works, but the screws are not available).
• Create a method to pick any entry point on the vertebra, not only the nodes. In fact, in this algorithm, the surgeon chooses the entry point, which must match one of the existing nodes of the STL model of the vertebra. If the vertebra is characterized by a mesh with lots of nodes, this problem disappears.
• Develop an application to be used on computers and laptops, so that the algorithm can be used in an X-Ray room or operating room.

## Ethical approval

All procedures were performed in accordance with the ethical standards of the institutional and/or national research committee. These procedures were in line with the 1964 Helsinki Declaration and its later amendments or comparable ethical standards.

Italian ethical approval: All procedures were performed in accordance with the ethical standards of current Italian law and of University of Salerno (Italy); the original study on the use of the custom-made templates was approved by the Ethical Review Board of the San Giovanni di Dio e Ruggi di Aragona University Hospital in Salerno, where the trial/tests were conducted. The trial itself and the proposed procedure have been approved by the Ethical Committee of Italian Health Ministry (Campania regional district) n.1, data February 01, 2017. The principal investigator, responsible for the clinical trial was Dr. Nicola Narciso, co-author of the paper [26] in references.

Informed consent: Medical data coming from real patients are related to participants that were informed about the testing protocol, the nature of the medical implements used and risks and benefits linked to the new surgical device. Informed consent was obtained in all instances.

## Data availability statement

Full data set about the template's Clinical trial is available publicly on demand at Italian Health Ministry, but not sharable publicly. Data related to the simulated tests on real patients are available on request because, even if the informed consent has been signed and data have been anonymized, the Italian law doesn't allow to share publicly the data set. Nevertheless, the specific data and data related to non-real patients are fully available on demand.

## CRediT authorship contribution statement

**Alfonso Magliano:** Writing – original draft, Validation, Software, Conceptualization. **Francesco Naddeo:** Writing – review & editing, Resources, Data curation. **Alessandro Naddeo:** Writing – review & editing, Supervision, Project administration, Methodology, Funding acquisition, Conceptualization.

## Declaration of competing interest

The authors declare that they have no known competing financial interests or personal relationships that could have appeared to influence the work reported in this paper.

## Appendix

The modules used in the Python programming environment are.

• *datetime*: Python built-in module. It is used to check how long the algorithm lasts.
• *vtk*: used for manipulating and displaying scientific data [49].
• *pyvista*: used for 3D plotting and mesh analysis through a streamlined interface for the Visualization Toolkit (VTK). It improves the vtk module performance [30].
• *numpy*: used for basic and complex scientific computing [50].
• *trimesh*: used to better manage and analyse the mesh (3.9 and 3.18) [31].
• *math*: Python built-in module. It provides access to the mathematical functions defined by the C standard.
• *subprocess*: Python built-in module. It allows you to spawn new processes, connect to their input/output/error pipes, obtain their return codes: it is used to let Python interact and communicate with MeshLab (3.3) and the notepad (3.3 and 3.28).
• *pandas*: used for the comparisons' realization [51] (3.24).
• *warnings*: Python built-in module used to suppress warnings. When the code checks if the found screw intersect the half vertebra (3.18), warnings of non-intersection will be displayed. This could cause, in some cases, algorithm's failure and stop. To avoid this problem, all the warnings are hidden.

## Footnotes

1 Image taken from Ref. [2].
